# Unravelling the interaction between *α*-SOH and myofibrillar protein based on spectroscopy and molecular dynamics simulation

**DOI:** 10.1016/j.fochx.2023.100986

**Published:** 2023-11-08

**Authors:** Jie Zhao, Shuaiqian Wang, Diandian Jiang, Yan Lu, Yu Chen, Yong Tang, Jie Tang, Zhenju Jiang, Hongbin Lin, Wei Dong

**Affiliations:** a School of Food and Bioengineering, Xihua University, Chengdu 610039, China; b Beijing Laboratory of Food Quality and Safety/Key Laboratory of Alcoholic Beverages Quality and Safety of China Light Industry, Beijing Technology and Business University, Beijing 100048, China; c Chongqing Key Laboratory of Speciality Food Co-Built by Sichuan and Chongqing, Chengdu 610039, China

**Keywords:** Myofibrillar proteins, Hydroxy-*α*-sanshool, Interaction mechanism, Spectroscopy, Molecular dynamics simulation

## Abstract

This work systematically investigated the dose–response interaction between hydroxy-*α*-sanshool (*α*-SOH) and pork myofibrillar proteins (MPs) via spectroscopy, molecular docking, and molecular dynamics simulation methods. Results showed that MPs bound with low *α*-SOH can enhance the surface hydrophobicity and particle size of MPs, whereas high concentrations were exactly the opposite. The main interaction force in *α*-SOH/MPs complex changed from hydrophobic to hydrogen bonding with increased *α*-SOH. *α*-SOH causes tryptophan quenching and bring about a red shift at low concentration, as well as to promote *α*-helix conversion into *β-*sheet in MPs. Simultaneously, molecular docking and dynamics simulations verified that hydrogen bonding and hydrophobic forces were the main contributors to *α*-SOH/MPs complex, indicating that the binding of *α*-SOH with MPs proceeded spontaneously with high intensity, in which TYR286 contributed the most significant energy. Therefore, revealing the binding mechanism of *α*-SOH and MPs can contribute to the deep processing of numbing meat products.

## Highlights

• *α*-SOH reduced the fluorescence of MPs by combining quenching.
• The binding of *α*-SOH to MPs promoted *α*-helix convert into *β-*sheet in MPs.
• Myosin TYR286 amino acid residue has the lowest binding energy to α-SOH.

## Graphical abstract

**Figure:**
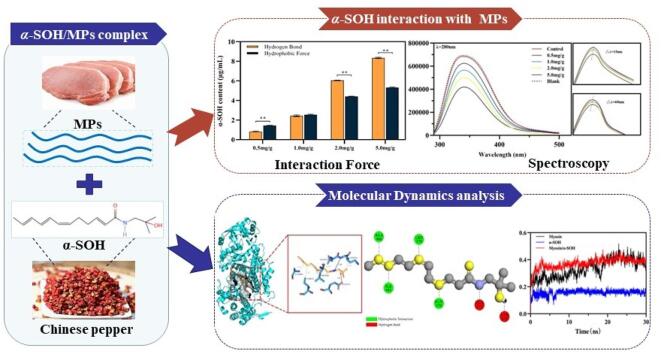

## 1 Introduction

Chinese pepper, the pericarps of fruits of *Zanthoxylum bungearnum Maxim*, is commonly used as the spice in Sichuan cuisine due to its unique sensation of tingling and numbing (Oh & Chung, 2014). The numbness of Chinese pepper is primarily attributed to sanshool, a chain-like unsaturated fatty amide compound, in which hydroxy-*α*-sanshool (*α*-SOH) with the highest content provides a robust stimulating sensation (Luo et al., 2022b). The molecular structure of *α*-SOH comprises the “head” vanilla group, the “neck” amide group, and the “tail” fatty chain. Differences in numbness are primarily related to the “head“ region, whereas the amide group in the “neck” acts as a flavor group and provides a site for attachment onto the receptor (Sun et al., 2020, Luo et al., 2022).

Current research on pepper numbing substances has focused on isolation and extraction and biological activity. Researchers have studied more than 30 kinds of unsaturated fatty acid amides by various purification and identification methods. Sanshool amide is confirmed to have significant antibacterial and antioxidant effects (Luo et al., 2022b). However, little attention has been paid to the flavor presentation mechanism of pepper sanshool amide in food.

Chinese pepper often acts as an aroma enhancer in Sichuan cuisine, especially for meat dishes. For these dishes, the numbing sensation primarily depends on the sanshool compounds that bind with meat proteins. Myofibrillar proteins (MPs), as the major proteins in muscle, are largely responsible for the flavor characteristics of meat products (He et al., 2021, Wang et al., 2022b), which can interact with flavor compounds through special molecular bonds to affect the flavor intensity and perception of meat products (Shen et al., 2020). The irreversible covalent linkages and reversible physicochemical binding (such as hydrogen bonds, van der Waals forces, hydrophobic forces, and electrostatic interactions) play important roles in the protein-flavor binding patterns (Shen et al., 2019a). Zhan et al. (2020) found that hydrophobic interactions play a vital role in the formation of the capsaicin-*β*-lactoglobulin complex. Researchers have found that aldehydes with longer carbon chains have a stronger combining capacity to proteins (Shen et al., 2019a). Meanwhile, the hydrophobicity and sulfhydryl content of myosin may alter the binding ability of volatile flavor constituents (Xu et al., 2020). Liu et al. (2022) found that fluorescence quenching on myosin increases with increased concentration of nonanal. Han et al. (2018) found that absorption intensity increases with increased concentration of nonanal in the UV–Vis absorption spectra of MPs, suggesting that different concentrations of nonanal can affect protein conformation. Investigating the interaction between protein and flavor compounds can facilitate food producers to develop protein based products with desirable flavor attributes. Among such flavors, the impacts of nonvolatile substances are of special considerations due to their practical contributions to meat products. Nevertheless, few studies have focused on the impact of *α*-SOH in Chinese pepper on protein-binding capacity.

In the present study, a complex containing various amounts of *α*-SOH and MPs was prepared to elucidate their interaction and primary intermolecular forces. The binding sites, interaction forces, and binding energies of *α*-SOH and MPs were simulated and validated by molecular docking and dynamics. Conformational changes of MPs upon *α*-SOH addition were also monitored to evaluate the potential correlation between the contents of *α*-SOH and their interaction characteristics with MPs.

## 2 Materials and methods

### 2.1 Materials

Porcine muscle *longissimus thoracis* (LT) was purchased from a local market (Chengdu, China). *α*-SOH was purchased from Macklin Biochemical Co., Ltd. (Shanghai, China). Urea, dithiothreitol (DTT), thiourea, CHAPS, and Triton X-100 were purchased from Aladdin Chemical Co., Ltd. (Shanghai, China). Protein-electrophoresis precast gels were purchased from Beyotime Biotechnology Co., Ltd. (Shanghai, China). All other chemicals used were analytical grade.

### 2.2 Sample preparation

#### 2.2.1 Preparation of myofibrillar proteins (MPs)

MPs were extracted from fresh pork LT as described by Zhao et al. (2022) with slight modifications. After being extracted with 10 mM phosphate buffer (PBS; containing 0.1 M NaCl, 2 mM MgCl_2_, 1 mM EDTA-2Na, pH 7.0), the protein was stored at 4 °C and used within 48 h. Biuret method was applied to determine the protein concentrations with bovine serum albumin as a standard.

#### 2.2.2 Preparation of complex

An appropriate amount of *α*-SOH powder was dissolved in 30 % ethanol to obtain stock solutions (mg/mL). MPs (mg/mL) were suspended in 0.1 M PBS buffer (pH 7.0) at room temperature. *α*-SOH/MP complexes were generated by blending an appropriate volume of *α*-SOH stock solutions and MP suspensions together to a final concentration of 0 (30 % ethanol as control), 0.5, 1, 2, and 5 mg/g (*m α*-SOH: *m* MPs). The mixture was stirred for 1 h at 200 rpm against light and incubated at 4 °C for 16 h.

### 2.2 Surface hydrophobicity (H_0_)

The sample’s H_0_ was determined following the method of Shi et al. (2022) with slight modifications. In a typical procedure, l mL of MP solution (5 mg/mL) or phosphate buffer (0.1 M, pH 7.0) was mixed well with 200 μL of bromophenol blue solution (BPB) and left at room temperature for 10 min before centrifugation (4000 *g*, 15 min). The absorbance of 595 nm was recorded, and the amount of BPB bound was calculated as follows:

$$BPBbound\left(\mu g\right)=200\mu g\times \left(A_{\mathrm{blank}}-A_{\mathrm{sample}}\right)/A_{\mathrm{blank}}$$

### 2.3 Particle size and zeta potential

The particle size and zeta potential of the samples (0.2 mg/mL) were measured using a laser particle-size analyzer (ZEN3600, Malvern Instruments Co., Ltd., UK) with phosphate buffer (10 mM, 0.1 M NaCl, pH 7.0) as a dispersion medium. For particle size, the relative refractive index and absorption were set as 1.333 and 0.001, respectively (Shen et al., 2019b). For zeta potential, 0.75 mL of solution was pipetted into the cuvette and measured at 25 °C.

### 2.4 Sodium dodecyl sulfate–polyacrylamide gel electrophoresis (SDS-PAGE)

The samples (2 mg/mL) were added to reducing (with DTT) and non-reducing buffer (without DTT) at a ratio of 1:4 (*v*/*v*) and boiled for 5 min before centrifugation (12000 *g*, 5 min). The supernatant was loaded onto a gradient gel of 4 %–20 % Tris-glycine (Tris-Gly) and electrophoresed at a constant current of 25 mA. The gels were stained with Coomassie brilliant blue R-250 for 30 min and decolorized with 10 % (*v/v*) glacial acetic acid and 10 % (*v*/*v*) ethanol until the background was colorless.

### 2.5 Sulfhydryl content

Total sulfhydryl content was measured according to the method of Amiri et al. (2018) with slight modifications. About 0.5 mL of MPs sample (5 mg/mL) was blended with 4.5 mL of Tris-Gly buffer (0.086 M Tris, 0.09 M glycine, 4 mM EDTA, 8 M urea, pH 8.0) and added to 0.5 mL of Ellman's reagent. The mixture was incubated at 25 °C for 30 min before recording the absorbance of 412 nm. Tris-Gly buffer without urea was applied to measure the free sulfhydryl content.

### 2.6 Free sanshool content

The standard curve of *α*-SOH was plotted based on a wide range of concentration from 0 μg/mL to 25 μg/mL at 254 nm absorbance. After incubating *α*-SOH/MP complexes with 8 M urea and 2 M thiourea before centrifugation (4000 *g*, 5 min) (Wang et al., 2022b), free *α*-SOH content was measured according to the standard curve to characterize the hydrogen bond and hydrophobic interaction in the complex.

### 2.7 Fluorescence measurements

#### 2.7.1 Intrinsic fluorescence spectroscopy

The intrinsic fluorescence of samples (0.5 mg/mL) was analyzed with a fluorescence spectrophotometer (Fluormax-4 HORIBA) at an excitation wavelength of 280 nm. The slits and emission spectral range was set to 5 nm and 280 to 500 nm, respectively.

#### 2.7.2 Fluorescence quenching

The assay solutions were prepared by mixing 0.8 mL of MP solution (5 mg/mL) with 500 μL of *α*-SOH solution, followed by adjusting the total volume to 10 mL with phosphate buffer (10 mM, 0.1 M NaCl, pH 7.0). The *α*-SOH concentrations were 0, 0.760, 1.520, 2.282, 3.041, and 3.802 × 10^–4^ M. The complexes were thoroughly mixed and incubated at different temperatures (298, 304, and 310 K) for 40 min prior to measurements. The fluorescence-quenching results were assessed by the Stern–Volmer equation (Baba et al., 2021):

$$\frac{F_{0}}{F}=1+K_{q}\tau_{0}\left[Q\right]=1+K_{\mathrm{sv}}\left[Q\right]$$

where F_0_ and F are the fluorescence intensities in the absence and presence of the quencher, respectively; Kq is the bimolecular quenching constant τ_0_ is the lifetime of the fluorophore in the absence of the quencher; Ksv is the Stern–Volmer quenching constant; and [Q] is the concentration of the quencher. Therefore, Ksv can be determined by applying linear regression to a plot of F_0_/F versus [Q].

The double logarithmic equation can be used to calculate n, which refers to the number of binding interaction sites in the *α*-SOH/MPs system, and equilibrium constant Ka for static quenching as follows (Jahanban-Esfahlan & Panahi-Azar, 2016):

$$log\frac{F_{0}-F}{F}=\;logK_{a}\;+\;nlog\left[Q\right]$$

To confirm the force of action between *α*-SOH and MPs, the thermodynamic parameters (ΔG, ΔH, and ΔS), contingent on temperatures, were calculated using the following Van’t Hoff equation and thermodynamic equation (Paiva et al., 2020):

$$lnK_{a}=\;-\frac{\Delta H}{RT}+\frac{\Delta S}{R}$$

ΔG = ΔH – TΔS.where the gas constant of R is 8.314 J/(mol·K).

#### 2.7.3 Synchronous fluorescence spectroscopy

Synchronous fluorescence can reflect changes in microenvironment polarity around fluorophores. Herein, spectra were acquired by simultaneously scanning the excitation and emission monochromators, with a constant wavelength interval of Δλ = 15 and 60 nm to reflect the microenvironment near Tyr and Try residues, respectively.

### 2.8 Circular dichroism (CD) measurements

CD spectra were acquired using a Chirascan System (Applied Photophysics Co., Ltd., Leatherhead, UK) with a 0.1 cm quartz cell and an average of three scans per spectrum within the range of 180–250 nm. The concentration of MPs and *α*-SOH/MP complexes were adjusted to 0.2 mg/mL and analyzed at a scanning speed of 120 nm/min and a scanning interval of 1 nm at 20 °C with nitrogen as the protective gas (Shen et al., 2020). CDNN software was used to calculate the protein secondary structure content.

### 2.9 Molecular docking

The amino acid sequence of myosin was obtained from UniProtKB (https://www.UniProt.org/) using genebank accession number P12883. The homology model was constructed using the online server (https://swissmodel.expasy.org/) with accession code 5TBY. The 3D structure of *α*-SOH was obtained from the PubChem database (PubChem CID: 10084135). Docking analysis was conducted with Autodock Vina software (version 1.1.2, Scripps Research Institute, La Jolla, CA, USA). A grid box with a size of 50 × 50 × 50 points covered all residues in the active site and centered on the following coordinates: X, −14.222; Y, 7.306; Z: −21.306.

### 2.10 Molecular dynamics (MD) simulations

Simulations were performed using the MD software GROMACS 2020.6. Proteins were generated in the AMBER99SB-ILDN force field with the corresponding parameterization file. SPC/E water model molecules were added to the center of the complex. Na^+^ ions were added to the complex box to maintain the electrical neutrality of the simulated system. Afterwards, a 50 000-step energy-minimization process was conducted by the steepest energy descent method. The temperature was maintained at 298.15 K using the Berendsen heat-bath method under NVT conditions and simulated for 50 000 steps (with each step lasting 2 fs) after performing energy minimization. Parrinello–Rahman with NPT was adjusted to 1 barometric pressure and simulated in 50 000 steps (2 fs per step). Finally, a 30 ns MD simulation (50 000-step, 2 fs each step) was conducted.

### 2.11 Statistical analysis

Statistical analysis was performed using SPSS 24 (IBM Inc., USA) software for ANOVA and Tukey's test for multiple comparisons of significant differences with *P* < 0.05. Data are exhibited as the mean ± standard deviation.

## 3 Results and discussion

### 3.1 Surface hydrophobicity

**Fig. 1:**
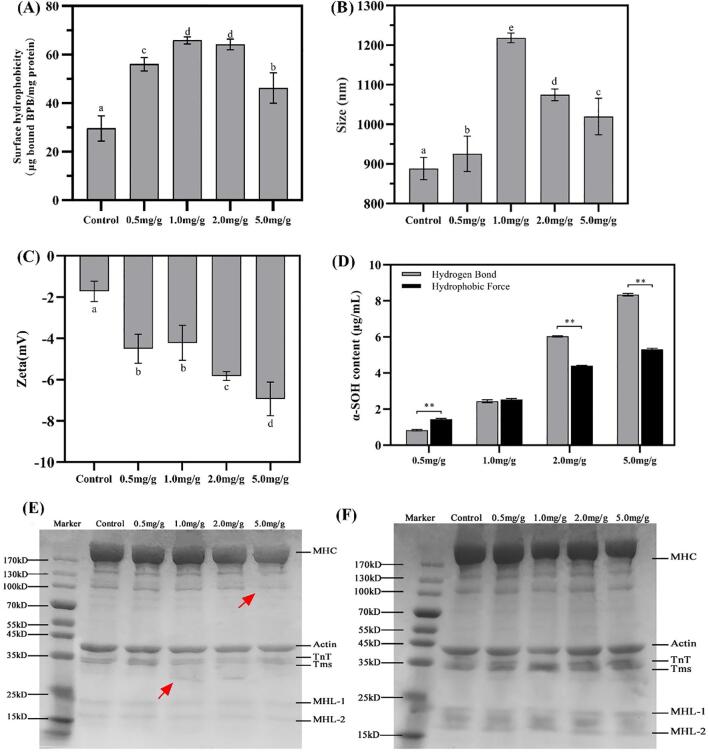
(A) Surface hydrophobicity of MPs and *α*-SOH/MPs complexes. (B) Particle size and (C) zeta potential of MPs and *α*-SOH/MPs complexes with different *α*-SOH concentrations. (D) Free *α*-SOH content of *α*-SOH/MPs complexes with bond disrupting agent solutions. The hydrogen bonding group indicated 8 M urea was added and the hydrophobic force group indicated 2 M thiourea was added (** *P*＜0.01). (E) Non-reduced (without DTT) and (F) reduced (with DTT) gel electrophoresis plots of MPs and *α*-SOH/MPs complexes. MHC, TnT, Tms, MHL-1, MHL-2 represent Myosin heavy chain, troponin T, tropmyosins, Myosin light chain-1, Myosin light chain-2, respectively.

H_0_ can provide some conformation changes of protein (Zhan et al., 2020). As shown in Fig. 1A, the H_0_ of *α*-SOH/MP complexes initially presented a significant increase with increased *α*-SOH concentration but decreased significantly when the *α*-SOH concentration reached 5.0 mg/g. The increase in H_0_ may be attributed to the noncovalent binding of *α*-SOH to MPs, which made the surface of the MPs more nonpolar (Jia et al., 2017). The nonpolar long carbon chain in *α*-SOH made it highly hydrophobic (Sun et al., 2020), which can combine with MPs via hydrophobic interaction forces to gain the hydrophobicity of complex. The decrease in H_0_ may be caused by several factors. On one hand, the hydrophobic force caused MPs to aggregate when *α*-SOH reached a high concentration, which can shield the hydrophobic region of MPs. On the other hand, hydrogen bonds formed between *α*-SOH and MPs, which enhanced the surface hydrophilicity and intermolecular forces of complex. This was determined by the amide group of *α*-SOH, which had a strong polar N—H bond and can form hydrogen bonds with other polar groups (Liang et al., 2020).

### 3.2 Particle size and zeta potential

The size of *α*-SOH/MP complex and the state of aggregation may be assessed by particle-size distribution (Han et al., 2022). Fig. 1B shows that the particle size of *α*-SOH/MP complexes initially increased and then decreased with increased *α*-SOH. The maximum particle size (1218.33 ± 87.75 nm) was reached at a concentration of 1.0 mg. Interestingly, the particle size was consistent with the results of H_0_, so we speculated that hydrophobic forces were one of the main reasons for the variation in particle size of the *α*-SOH/MP complex. The protein cross-linking caused by amide groups also cannot be ignored. A study on glutamine transaminase cross-linked proteins has shown that protein cross-linking is achieved by acyl transfer between the *γ*-carboxyamide groups of glutamine residues and *ε*-amino groups of lysine residues (Shi et al., 2022). Similarly, protein cross-linking caused by covalent binding between disulfide bonds is not yet verified. Zeta-potential results (Fig. 1C), revealed that the potential values consistently increased, inconsistent with the trend of H_0_ and particle size. This finding indicated that a covalent or noncovalent force was greater than the electrostatic force and dominated at high concentrations of *α*-SOH.

### 3.3 SDS-PAGE

Reduction (+DTT) and nonreduction (–DTT) electrophoresis experiments were performed to examine the interaction force in protein cross-linking. Under the –DTT condition (Fig. 1E), the 35 kDa band weakened and was followed by a slight enhancement in the band at around 30 kDa in the 1.0 and 2.0 mg/g groups compared with the control and 0.5 mg/g groups. When the *α*-SOH concentration reached 5.0 mg/g, the 100 and 130 kDa bands significantly weakened. Thus, some subunits of MPs were degraded at > 1.0 mg/g, most obviously in the myosin heavy chain (MHC), troponin T, (TnT), and tropmyosins (Tms). Mun et al. (2014) found that under low Ca^2+^ conditions, myosin-binding protein C (MyBP-C) was able to compete with tromyosins for the actin-binding head at the same binding site. Meanwhile, tromyosins moved to high Ca^2+^. Therefore, the increase in *α*-SOH concentration may alter the ion concentration in the system, which can disrupt the cross-linking of myosin and cause its degradation. Combining the particle size and surface hydrophobicity analysis indicated that the degradation of the subunits at 1.0 mg/g allowed the exposure of more hydrophobic groups, which caused the protein to aggregate into larger particles due to the hydrophobic forces. Under the + DTT condition (Fig. 1F), the myosin light chains near the 15–24 kDa band significantly deepened. This phenomenon proved that the disulfide bonds in MPs were cleaved by DTT, which increased the content of small-molecule proteins, but the protein multimerization state in MPs was not destroyed by *α*-SOH addition. A similar phenomenon was observed by Zhan et al. (2020) in their study on the interaction of capsaicin with *β*-lactoglobulin.

### 3.4 Total and free sulfhydryl content

Total sulfhydryl represents all sulfhydryl groups on the surface and internal regions of the protein network, whereas free sulfhydryl refers to sulfhydryl groups exposed on the surface of the protein network (Wang et al., 2017). Compared with MPs, the total sulfhydryl content of the *α*-SOH/MP complexes slightly increased (Table 1), which may be attributed to the fact that the conjugated triple bond in *α*-SOH structure is highly sensitive to oxygen. Thus *α*-SOH acted a reducing agent to some extent (Kolodiazhna & Kolodiazhnyi, 2019). Nevertheless, no significant variation was observed among the *α*-SOH/MP complexes with different concentrations of *α*-SOH. Similar results were obtained in free sulfhydryl groups. This result indicated that no disulfide bonds formed between *α*-SOH and MPs, and all sulfhydryl groups were carried by the cysteine of MPs. This finding was due to the attachment of the amino nitrogen in the amide group onto the aliphatic alkyl group (RCH_2_^–^), which affected the electron repulsion force in amino nitrogen. Consequently, the electron-cloud density of the nitrogen atom increased and the nitrogen with lone pairs of electrons difficultly combined with other atoms to form covalent bonds (Kuroki et al., 2016).

**Table 1:** The quenching rate constants (Ksv), correlation coefficients (Kq), correlation coefficients (R_1_), apparent binding constant (Ka), binding site number (n), and correlation coefficients (R_2_) of *α*-SOH/ Myosin at 293, 303, 310 K. (values are means ± SD).

Temperature (K)	293 K	303 K	310 K
Ksv (10^3^ M^−1^)	0.75 ± 0.06a	1.12 ± 0.11b	1.46 ± 0.08c
Kq (10^11^M^-1^s^−1^)	0.75 ± 0.06a	1.12 ± 0.11b	1.46 ± 0.08c
R_1_^2^	0.9877	0.9881	0.9941
Ka (10^2^M^−1^)	12.65 ± 0.12c	0.33 ± 0.01a	0.93 ± 0.04b
n	1.482	0.7936	0.9184
R_2_^2^	0.9754	0.9907	0.9984
△H (kJ/mol)	−164.076		
△S (kJ/mol)	−0.501		
△G (kJ·mol^−1^·K^−1^)	−17.283	−12.273	−8.766

### 3.5 Free sanshool content

After the above experiments, the contribution of covalent linkages in the binding of *α*-SOH to MPs was largely ruled out. Accordingly, we first focused on the hydrogen bond and hydrophobic forces (the main forces in noncovalent interactions) in the contribution of binding forces. Urea significantly contributes to breaking hydrogen-bond forces, whereas thiourea is more inclined to break hydrophobic forces (Wang et al., 2022a). After incubation with the bond-blocking agent, the contribution of noncovalent forces can be determined by measuring the amount of free *α*-SOH in the supernatant. Fig. 1D shows that the hydrophobic contribution was greater than the hydrogen bond in the 0.5 mg/g group, and no significant discrepancy existed for the 1.0 mg/g group. Conversely, the hydrogen-bond contribution was greater than the hydrophobic contribution for the 2.0 and 5.0 mg/g groups. Thus, the contribution of noncovalent forces may shift with increased *α*-SOH concentration. The binding force was primarily contributed by hydrophobic interactions at low concentrations (<1.0 mg/g) of *α*-SOH but was converted to hydrogen bonding at high concentrations (>2.0 mg/g). The number and arrangement of the amide groups reportedly play important roles in the properties of the solutions (Kakehashi et al., 2012). With increased *α*-SOH concentration, the number of amide groups and the alignment length increased, thereby effectively promoting hydrogen-bond formation between *α*-SOH and MPs.

### 3.6 Fluorescence measurements

#### 3.6.1 Intrinsic fluorescence and synchronous fluorescence spectroscopy

**Fig. 2:**
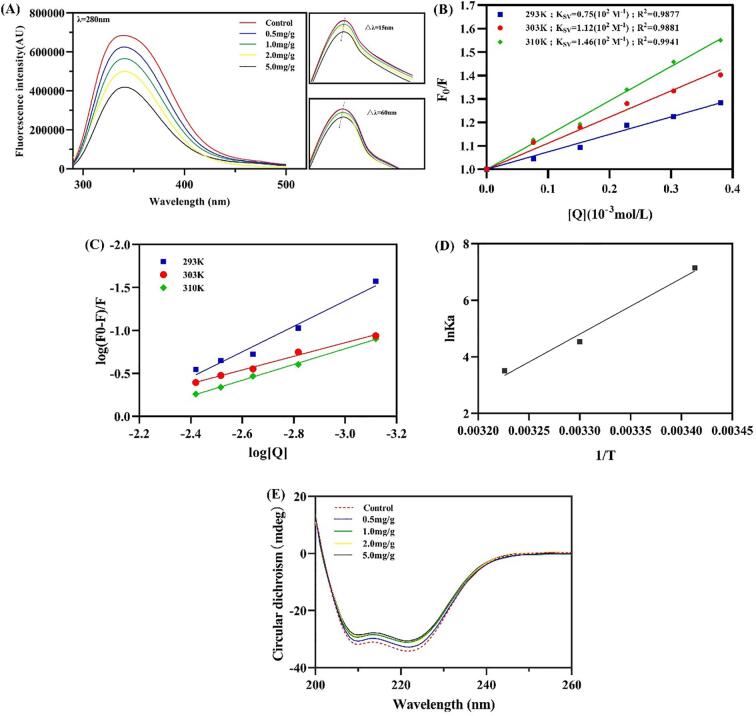
(A) Intrinsic fluorescence (note λ = 280 nm) and synchronous fluorescence (note △λ = 15 nm and 60 nm) spectra of MPs and *α*-SOH/MPs complexes. Blank group indicated that the same amount of ethanol solvent was added to the MPs as the 5.0 mg/g group. (B) Sterne-Volmer plot, (C) Double logarithmic plot and (D) Van't Hoff plot of interaction between *α*-SOH and MPs at different temperatures. (E) Circular dichroism of MPs and *α*-SOH/MPs complexes. (F).

As shown in Fig. 2A *α*-SOH can act as a quencher, and the degree of fluorescence quenching of MPs increased with increased concentration. With increased *α*-SOH addition, the λ max of complex initially red shifted gradually from 337 nm (control) to 341 nm (2.0 mg) and then slightly blue shifted to 340 nm (5.0 mg/g). This result indicated an alteration in the polar environment of the amino acids (Liu et al., 2019), consistent with the trend of H_0_.

Synchronous fluorescence is used to determine the environmental changes of fixed fluorescent groups. The difference between excitation and emission wavelengths (Δλ) of 15 and 60 nm can characterize the Tyr and Trp residues, respectively. The emission peaks exhibited a blue shift from 315 nm to 313 nm at Δλ = 15 nm and from 357 nm to 355 nm at Δλ = 60 nm. This finding demonstrated that the environment around Tyr and Trp was less polar and more hydrophobic and that Tyr and Trp can be further encapsulated in the inner region of the protein

#### 3.6.2 *Fluorescence* quenching of MPs by *α*-SOH

*α*-SOH had a fluorescence quenching effect on MPs, so a discussion about the mechanism of fluorescence quenching was initiated to determine the static and dynamic quenching based on the dependence on temperature (Wang et al., 2022a). Dynamic quenching is caused by collisions between two substances in the excited state, whereas static quenching is caused by the formation of a nonluminous ground-state complex between quencher and fluorophore (Shen et al., 2019a). The fitting plot and detailed data of *α*-SOH-quenching MPs are shown in Table 2 and Fig. 2B, respectively. The quenching constant (Ksv) increased with increased temperature, indicating a dynamic quenching in the interaction of *α*-SOH with MPs. However, the quenching rate constant (Kq) was greater than the maximum diffusive collision rate constant (2 × 10^10^ M^−1^s^−1^) (Shahabadi et al., 2015), suggesting that static quenching was also involved in the interaction. Thus, the process of *α*-SOH quenching the fluorescence of MPs was a form of combined quenching.

**Table 2:** Total and free sulfhydryl content (values are means ± SD).

	Control	0.5 mg/g	1.0 mg/g	2.0 mg/g	5.0 mg/g
Total -SH	29.30 ± 0.07a	30.04 ± 0.07ab	30.56 ± 0.63b	30.12 ± 0.26b	30.11 ± 0.03b
Free -SH	9.14 ± 0.17a	9.75 ± 0.01b	9.64 ± 0.01b	9.76 ± 0.02b	9.74 ± 0.03b

The number of binding sites (n) and the binding constant (Ka) can reflect the binding affinity. Fig. 2C shows the plot made after fitting the double-logarithmic equation to calculate the binding site n and Ka. The n value was close to 1 at 310 K, and Ka remained in the order of 10^2^ M^−1^; however, it showed signs of decreasing with increased temperature. This finding indicated general ability to combine the *α*-SOH/MP complex.

The values of ΔH, ΔS, and ΔG were calculated by combining the thermodynamic equations (Fig. 2D and Table 1). Negative values of ΔG indicated that the interaction of the system was spontaneous. The values of enthalpy (ΔH) and entropy (ΔS) can be used to determine the force driving the interaction (Paiva et al., 2020). For the *α*-SOH/MP complex, the values of ΔH and ΔS were −164.076 and −0.501 kJ/mol, respectively, illustrating that hydrogen bonding was an important force that drove the binding of *α*-SOH with MPs.

### 3.7 Circular dichroism (CD) analysis

The CD spectrum of sample showed two negative peaks at 208 and 222 nm (Fig. 2E) due to the *α*-helical structure in the myosin tail (Chen et al., 2019). After data processing, the *α*-helix content was found to decrease from 15.42 % to 13.77 %, whereas *β*-sheet increased from 30.58 % to 32.55 % with increased *α*-SOH concentration. This finding was due to *α*-SOH possibly attacking the amino acid residues of the MPs and disrupting the hydrogen bond in the *α*-helix, indicating that the secondary structure of the protein shifted from an ordered to a disordered state (Qi et al., 2018). In conjunction with a previous analysis on noncovalent forces, we speculated that the hydrogen bonds between amino acid residues within the protein molecule may have in turn bound with *α*-SOH, thereby disturbing the ordered structure of protein.

### 3.8 Homology modeling and molecular docking

**Fig. 3:**
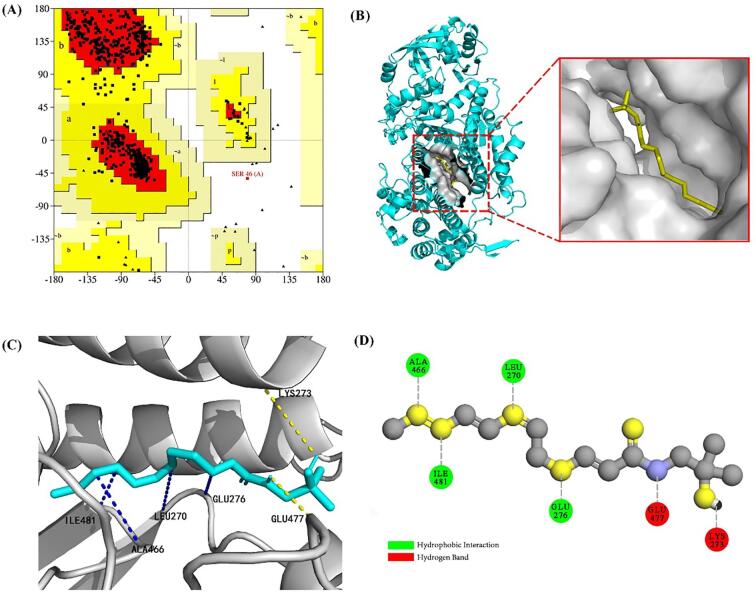
(A) Ramachandran plot for the homology model. (B) Overall view of the *α*-SOH/Myosin complex docking. (C) Partial view of the SOH/Myosin complex docking. (D) 2D diagram of a zoomed-in perspective of the *α*-SOH/Myosin complex.

Molecular docking enables the determination of the binding mode and the binding site between small molecules and proteins (Li et al., 2019). Considering that myosin is the major protein in MPs (about 55 %–60 %), myosin was chosen as the protein receptor for *α*-SOH in the homology modeling (Wang et al., 2022a). As shown in Fig. 3A, the Ramachandran plot obtained from the modeling showed that 95.66 % of the dihedral angles were in the permissible region and that most amino acids were within the error margin, showing that the model conformed with expectations. Fifty of the poses were docked in Autodock Vina, and the lowest energy group (-5.55 kcal/mol) was finally selected for docking analysis. Fig. 3B and C show the overall and local details of *α*-SOH docking with myosin, respectively. Fig. 3D shows a 2D view of the docking detail. As shown in Fig. 3D, the hydrogen bond and hydrophobic force were the main interaction forces in the complex, consistent with the conclusions of the above experiments. The amino acid residues ALA 466, ILE 481, LEU 270, GLU 276, and LYS 273 in myosin had a hydrophobic interaction with *α*-SOH, which was primarily located at the hydrophobic chain of the *α*-SOH tail. The GLU 477 and LYS 273 amino acid residues in myosin had hydrogen-bond interactions with *α*-SOH. The hydrogen bond was also found at the *N*-terminal end of the amide group and at the hydroxyl end of the *α*-SOH head, respectively. This finding was consistent with the theoretical position of the hydrogen bond (Ghosh, 2019).

### 3.9 Molecular dynamics (MD) simulation

**Fig. 4:**
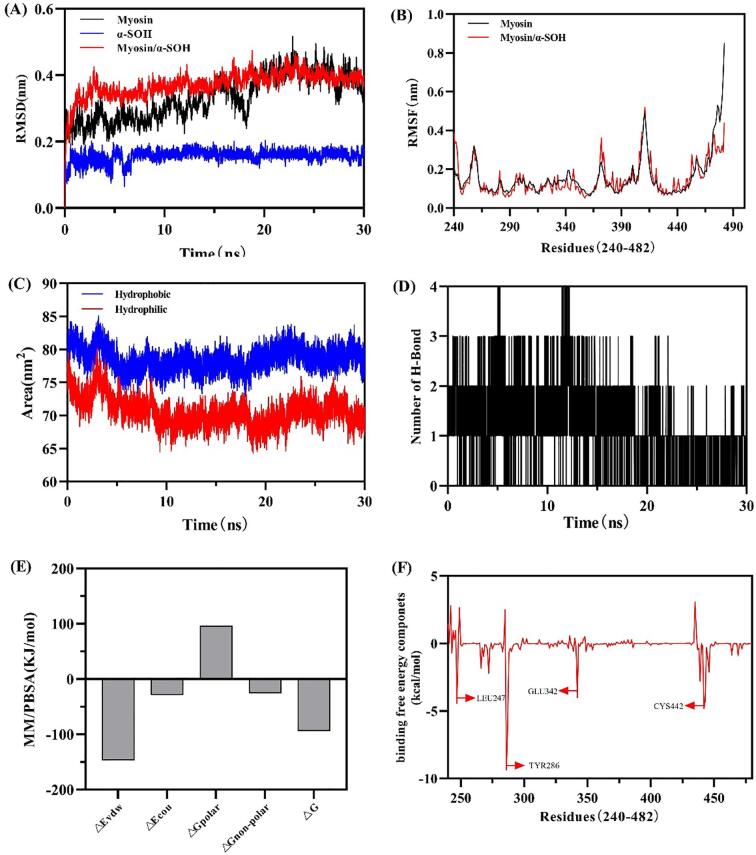
(A) The root mean square deviation (RMSD) values, (B) root mean square fluctuation (RMSF) values and (C) solvent accessible surface area (SASA) values of myosin and the *α*-SOH/Myosin complex. (D) The number of hydrogen bonds in the *α*-SOH/Myosin complex during the simulation time. (E) The Averaged binding free energies of the simulated *α*-SOH/ Myosin complex. △E_vdw_: van der Waals interaction energy; △E_cou_: electrostatic interaction energy; △G_polar_: Free energy of polarizing solvents; △G_non-polar_: free energy of non-polarizing solvents; △G: binding energy. (F) Contribution of single residues to the binding free energy of *α*-SOH/Myosin complex.

To further explore the changes in *α*-SOH and myosin, 30 ns of molecular dynamics simulations were performed. Root-mean-square deviation (RMSD) is often used to estimate the stability of the molecular structure, and a small RMSD value indicates a stabilization of the structure (Verma et al., 2021). The RMSD plot in Fig. 4A shows that the *α*-SOH/myosin complex considerably fluctuated from 0 to 4 ns and then stabilized. The average RMSD values for myosin and *α*-SOH/myosin complex were 0.3722 and 0.3322, respectively, suggesting that binding *α*-SOH with myosin had less effect on protein freedom. Root-mean-square fluctuation (RMSF) indicates the fluctuation of residues in a protein, with larger RMSF values signifying greater flexibility change (Yu et al., 2021). The mean RMSF values of the *α*-SOH/myosin complex (0.1509 nm) were lower than those of myosin (0.1563 nm) as shown in Fig. 4B. Thus, the binding of *α*-SOH inhibited the fluctuation of myosin, which was attributed to the stronger hydrogen bond between myosin and *α*-SOH than that within myosin. The *α*-SOH/Myosin complex also largely varied in amino acid structural flexibility within the regions of 257–290, 371–374, 406–416, and 453–547. Solvent-accessible surface area (SASA) is primarily used to measure the variation in the surface area of proteins over time, which can be divided into hydrophilic and hydrophobic. Fig. 4C shows that the hydrophobic SASA was higher than the hydrophilic SASA, and the values remained stable. This finding may be related to the attachment of the hydrophobic *α*-SOH onto the myosin surface. The number of hydrogen bonds between *α*-SOH and myosin is shown in Fig. 4D. Stable hydrogen bonds were always present during MD, indicating that hydrogen bonds significantly contributed to the stability of the *α*-SOH/myosin complex.

Binding free energy is used to evaluate the interaction between *α*-SOH and myosin (Farhadian et al., 2019). The free energy of binding *α*-SOH with protein was −94.072 ± 1.346 kJ/mol (Fig. 4E). The negative binding energy meant that binding can occur spontaneously with high strength. Fig. 4F shows the energy contribution of the major amino acid residues. The the most significant energy contribution was that of TYR286, followed by LEU247, CYS442, and GLU342.

## 4 Conclusion

This study investigated the interaction mechanism of *α*-SOH/MP complex. Results showed that the interaction between *α*-SOH and MPs may increase the H_0_ and particle size of the complex within a low concentration of *α*-SOH (<2 mg/g). This finding indicated that hydrophobic forces were one of the main reasons for the variation in particle size. Further investigation revealed that the primary binding force changed from hydrophobic to hydrogen bonding with increased *α*-SOH concentration and that no covalent linkage was involved. Spectroscopic results further indicated that *α*-SOH quenched the fluorescence of MPs primarily through a combined quenching procedure. The interactions with *α*-SOH increased the *β*-sheets at the expense of *α*-helices. *α*-SOH subsequently interacted with MPs primarily through the spontaneous reversible binding force of hydrophobic and hydrogen bonding. A notable amino acid residue was TYR286, which had the lowest binding energy to *α*-SOH. Revealing the binding mechanism of *α*-SOH and MPs can benefit the development of numbing meat products.

## CRediT authorship contribution statement

**Jie Zhao:** Conceptualization, Supervision, Writing – review & editing, Funding acquisition, Validation. **Shuaiqian Wang:** Methodology, Software, Investigation, Formal analysis, Writing – original draft. **Diandian Jiang:** Methodology, Software. **Yan Lu:** Writing – review & editing. **Yu Chen:** Methodology, Investigation. **Yong Tang:** Writing – review & editing. **Jie Tang:** Writing – review & editing. **Zhenju Jiang:** Writing – review & editing. **Hongbin Lin:** Writing – review & editing. **Wei Dong:** Resources, Supervision, Writing – review & editing.

## Declaration of Competing Interest

The authors declare that they have no known competing financial interests or personal relationships that could have appeared to influence the work reported in this paper.

## Data Availability

Data will be made available on request.
